# Patient and staff satisfaction with remote psychiatry assessments using mobile tablets in long-stay facilities in rural north-west Ireland

**DOI:** 10.1192/j.eurpsy.2021.1145

**Published:** 2021-08-13

**Authors:** S. Patel, E. Maye, A. Gannon, M. Cryan, C. Dolan, G. Mccarthy

**Affiliations:** 1 Psychiatry Of Old Age, Sligo/Leitrim Mental Health Services, Sligo, Ireland; 2 School Of Medicine, Cardiff University, Cardiff, United Kingdom; 3 Sligo Medical Academy, NUIG, Sligo, Ireland

**Keywords:** older persons, mobile tablet, psychiatry, nursing homes

## Abstract

**Introduction:**

The COVID-19 pandemic has required services to evolve quickly to continue routine care and telemedicine has been rapidly implemented to facilitate this. Older persons are at high risk of serious complications of COVID-19 and it is essential that their exposure to COVID-19 is minimized.

**Objectives:**

Our aim was to assess staff and patient satisfaction with remote psychiatric assessments using mobile tablets in long-stay facilities.

**Methods:**

Remote clinics using Skype video on mobile tablets were conducted with patients in long-stay facilities attending psychiatry in rural North-West Ireland between April and July 2020. At each review, a satisfaction survey was administered to the patient, their keyworker and the clinician. The patient/keyworker survey instrument had four yes/no statements and the clinician survey had four statements with 5-point likert scale responses (1=very low to 5=very high). Open feedback was also obtained for thematic analysis. Descriptive analyses were completed using SPSS software.

**Results:**

23 patients (mean age 80.9yrs) were assessed in 10 long-stay facilities. All patients were agreeable to participating in video consultation although only 13 patients were able to respond to survey due to cognitive impairment. There was a 92.3% positive patient response (12/13) and 95.7% positive keyworker response (N=22/23) for all statements. The mean score on the assessor response ranged from 3.43 to 4.04 with the lowest rate for quality of transmission. The main themes identified were related to the quality of connection and sensory difficulties.

**Conclusions:**

Video consultations using mobile tablets offer an acceptable form of remote psychiatry assessment for older persons in long-stay facilities.

